# 2D Titanium-Based Nanozyme Induced a Ferroptosis-like Strategy for Triple-Negative Breast Cancer Therapeutics

**DOI:** 10.34133/bmr.0400

**Published:** 2026-09-21

**Authors:** Hailing Yu, Songying Pi, Zebo Jiang, DongHui Huang, Xinxi Yu, Feile Ye, Ting Yu, Huimin Lan, Qianqian Zhang, Jingchuan Zhu, Lili Wu, ZhiSheng Nong, Yin Huang, Yongquan Huang

**Affiliations:** ^1^ Guangdong Provincial Engineering Research Center of Molecular Imaging, Guangdong-Hong Kong-Macao University Joint Laboratory of Interventional Medicine, The Fifth Affiliated Hospital, Sun Yat-sen University, No. 52 of Meihuadong Road, Xiangzhou District, Zhuhai, Guangdong, China.; ^2^ Department of Ultrasound, The Fifth Affiliated Hospital, Sun Yat-sen University, No. 52 of Meihuadong Road, Xiangzhou District, Zhuhai, Guangdong, China.; ^3^ Zhuhai Hospital of Integrated Traditional Chinese & Western Medicine, Zhuhai, Guangdong, China.; ^4^ School of Materials Science and Engineering, Harbin Institute of Technology, No. 92 of Xidazhi Street, Nangang District, Harbin, Heilongjiang, China.; ^5^ Key Laboratory for Photonic and Electronic Bandgap Materials, Ministry of Education, School of Physics and Electronic Engineering, Harbin Normal University, No.1 of Shida South Road, Limin Economic and Development District, Harbin, Heilongjiang, China.; ^6^ School of Materials Science and Engineering, Shenyang Aerospace University, No. 37 of Daoyinan Street, Shenbei New District, Shenyang, Liaoning, China.; ^7^ Center of Cardiovascular Disease, The Fifth Affiliated Hospital, Sun Yat-sen University, No. 52 of Meihuadong Road, Xiangzhou District, Zhuhai, Guangdong, China.

## Abstract

Ferroptosis, a regulated cell death pathway driven by iron-dependent lipid peroxidation, has emerged as a promising strategy for cancer therapy. Nevertheless, the direct delivery of iron can lead to systemic toxicity. In this study, a nonferrous ferroptosis-like strategy employing 2D Ti_3_C_2_ MXene nanosheets was developed. The Ti_3_C_2_ nanozyme has been shown to possess peroxidase- and catalase-like activities, capable of decomposing H_2_O_2_ into cytotoxic·OH and O_2_, respectively. When exposed to near-infrared II laser irradiation, Ti_3_C_2_ induced a localized increase in temperature and amplified reactive oxygen species production, resulting in a synergistic effect that led to mitochondrial damage and glutathione peroxidase 4 inactivation. In vivo studies demonstrated tumor suppression in triple-negative breast cancer (TNBC) models, accompanied by M1 macrophage polarization and CD8^+^ T cell activation. This work provides a novel paradigm for nanocatalytic ferroptosis therapy, combining enzymatic activity, photothermal effects, and immunomodulation to overcome TNBC resistance.

## Introduction

Breast cancer ranks as the most prevalent malignant tumor worldwide, with triple-negative breast cancer (TNBC) accounting for 15% to 20% of cases and exhibiting a dismal 5-year survival rate of just 12.2% in advanced stages [1,2]. Unlike other subtypes, TNBC cells display unique metabolic vulnerabilities, including dysregulated iron homeostasis and glutathione (GSH) balance, rendering them susceptible to ferroptosis, which is a nonapoptotic cell death driven by iron-dependent lipid peroxidation (LPO) [3,4]. While ferroptosis induction holds therapeutic potential, direct iron delivery risks systemic toxicity [5], and tumor antioxidant defenses often counteract reactive oxygen species (ROS)-mediated lethality [6,7].

Current strategies predominantly rely on iron-based nanomaterials to catalyze Fenton reactions, generating hydroxyl radicals (·OH) from H_2_O_2_ [8]. However, their efficacy is limited by tumor microenvironment (TME) constraints [9,10]. Moreover, excessive iron exacerbates oxidative stress in normal tissues [5]. Recent advances propose nonferrous metal-based nanozymes as alternatives, leveraging ROS amplification and GSH depletion to disrupt redox homeostasis [11,12]. Such approaches may concurrently stimulate antitumor immunity by modulating tumor-associated macrophage (TAM) polarization and dendritic cell maturation [13,14]. The development of nonferrous metals analogous to the concept of a ferroptosis-like effect may provide an alternative and effective strategy by integrating a nanoplatform that achieves maximum cell death through a synergistic ROS burst and GSH depletion.

Ti_3_C_2_ MXenes, characterized by their 2-dimensional (2D) structure, provide an optimal surface for catalytic site attachment, thereby facilitating the etching of the aluminum layer from a MAX-phase material [15]. In a manner analogous to that observed with iron-based nanomaterials, 2D Ti_3_C_2_ nanosheets demonstrate multiple enzymatic activities, including peroxidase (POD) activity, catalase (CAT) activity, and glutathione peroxidase (GPX) activity [16]. Moreover, previous studies have demonstrated that the introduction of external energy, such as laser irradiation, can effectively lower the activation energy barrier of metal cations, thereby potentially augmenting the catalytic efficiency of nanozymes [6]. Hence, near-infrared II (NIR-II) laser irradiation can be used as an exogenous stimulation focused on the tumor site, with the potential advantages of deep tissue penetration and precise spatiotemporal selectivity.

**Fig. 1.**
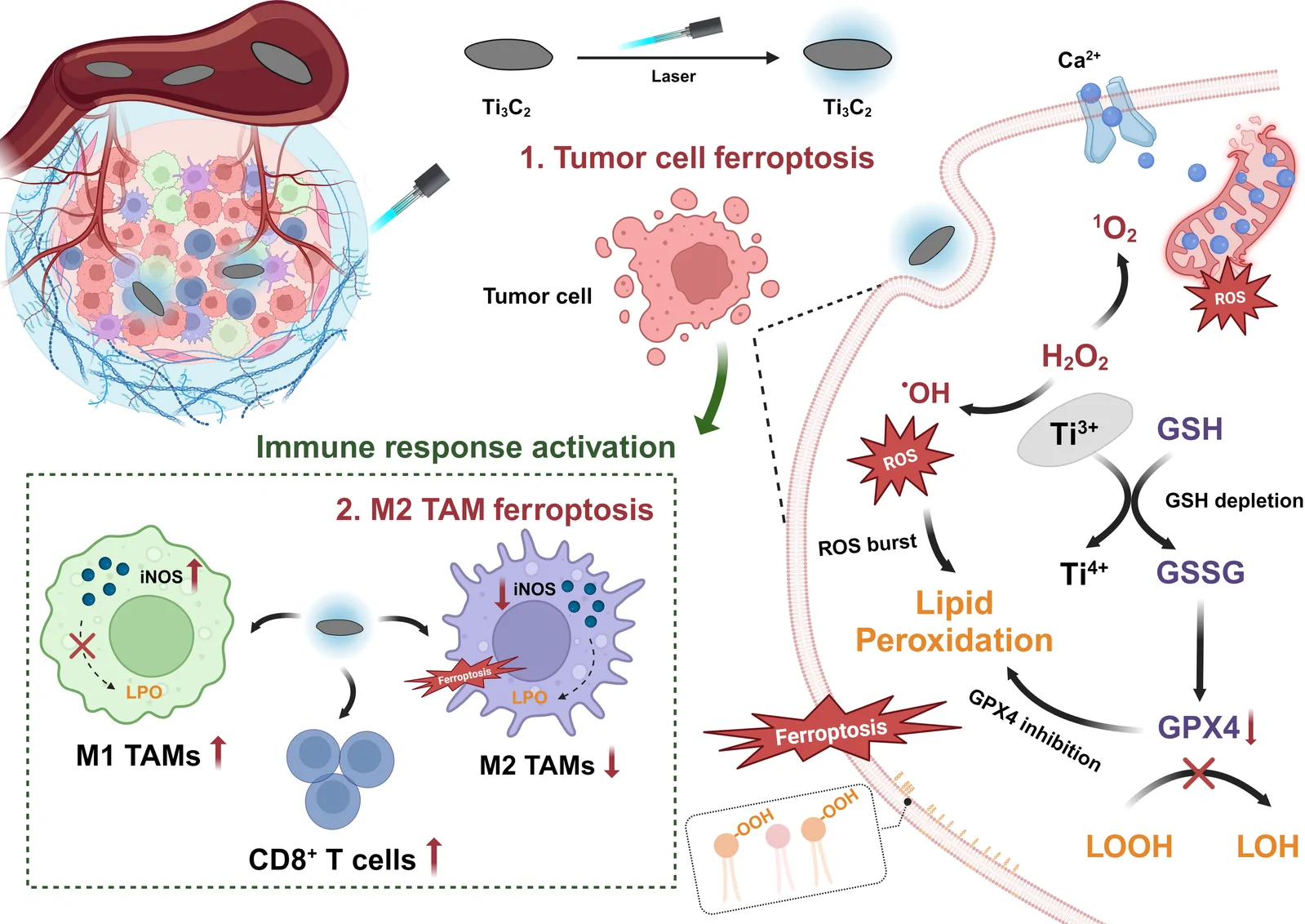
The schematic diagram of titanium-based nanozyme for immunomodulation-enhanced tumor therapy (created with BioRender.com).

In this study, we develop a nonferrous ferroptosis-like strategy using 2D MXene nanozymes to synergistically integrate nanocatalytic ferroptosis, photothermal therapy, and immune activation (Fig. 1). The strategy involves depleting GSH, down-regulating GPX4, and amplifying lipid peroxidation, thereby overcoming TNBC resistance. This multimodal strategy has the potential not only to directly eradicate tumor cells but also to reshape the immunosuppressive TME, thus offering a transformative approach for the treatment of TNBC.

## Materials and Methods

### Materials

The mouse breast cancer cells and normal human kidney cells were obtained from Jennio-bio (China). RPMI 1640 medium, fetal bovine serum, trypsin-EDTA, and penicillin-streptomycin were purchased from Gibco (USA). The live-dead cells kit (calcein-AM and propidium iodide) and the ROS indicator (dichlorofluorescin diacetate) were purchased from Beyotime Biotechnology (China).

### Synthesis of Ti_3_C_2_ nanosheets

To prepare Ti_3_C_2_, a mixture of lithium fluoride and hydrochloric acid solution was stirred thoroughly. Then, the Ti_3_AlC_2_ powder was added to the mixture and left at 4 °C for 24 h. The resulting precipitate was collected by centrifugation, washed with water, and stored at 4 °C.

### Characterization

Transmission electron microscopy (Talos F200i S) was used to obtain transmission electron microscopy and mapping images of Ti_3_C_2_. The crystal structure of Ti_3_C_2_ was captured using an x-ray diffractometer (Rigarke smartlab). Thermo 250XI spectrophotometers were used to study the x-ray photoelectron spectroscopy (XPS) spectra.

### Cell culture

Mouse breast cancer cells (4T1) and normal human kidney cells (293T) were purchased from the Jennio-bio (China). The cancer cells were cultured with RPMI 1640 medium containing 10% fetal bovine serum and 1% penicillin-streptomycin, while the normal cells were cultured with Dulbecco’s modification of Eagle’s medium containing 10% fetal bovine serum and 1% penicillin-streptomycin. Both cell types were incubated at 37 °C with 5% CO_2_.

### The cell viability in vitro

The cytotoxicity of Ti_3_C_2_ to cancer cells was studied using the Cell Counting Kit-8 (CCK8) assay. 4T1 cells were seeded in 96-well plates and treated with different concentrations of Ti_3_C_2_ for 24 h. A laser with a power of 1.5 W/cm^2^ was applied to the cells for 3 min. After removing the medium, fresh medium containing 10% CCK8 was added to the plates, and cell viability was measured using a multimode microplate reader (PerkinElmer EnVision 2105, USA). Live/dead cells were investigated using Calcein-AM/propidium iodide (PI) staining. Cancer cells were seeded in 24-well plates and exposed to Ti_3_C_2_ for 8 h before laser irradiation for 3 min. Calcein-AM/PI staining was added to the plates for 30 min and detected using confocal laser scanning microscopy (Carl Zeiss LSCM 880, Germany).

### Evaluation of ROS, LPO, Fe^2+^, and GSH in vitro

ROS, LPO, Fe^2+^, and GSH generation were analyzed using 2′,7′-dichlorodihydrofluorescein diacetate (DCFH-DA), Liperfluo, FerroOrange, and ThiolTracker Violet probes. 4T1 cells were seeded in 6-well plates and treated with various agents. After incubation with DCFH-DA, Liperfluo, FerroOrange, and ThiolTracker Violet probes for 20 min, the samples were imaged using confocal laser scanning microscopy.

### The evaluation of mitochondrial dysfunction in vitro

The study evaluated mitochondrial dysfunction in 4T1 cells using a JC-1 probe to detect changes in mitochondrial membrane potential. The cells were seeded into a 6-well plate and confocal dishes, and Ti_3_C_2_ was added to the medium for 8 h. The cancer cells were detected using flow cytometry (FCM) and confocal laser scanning microscopy under laser irradiation. Mito-tracker dye was used to evaluate mitochondrial dysfunction in 4T1 cells. The cells were treated with phosphate-buffered saline (PBS), laser, Ti_3_C_2_, and Ti_3_C_2_ plus laser (1.5 W/cm^2^, 3 min). After treatment, the cells were stained with Mito-tracker, and the nuclei with 4′,6-diamidino-2-phenylindole.

### Density functional theory calculation

To investigate the activation process of H_2_O_2_, we utilized the Cambridge Serial Total Energy Package code based on density functional theory (DFT) to calculate the reaction between H_2_O_2_ and Ti_3_C_2_. The ultrasoft pseudopotential was used to describe the electron-ion interaction, and the generalized gradient approximation with Perdew–Burke–Ernzerhof parameters was employed to describe the exchange and correlation terms. The calculation utilized cutoff energy of 380 eV, and the appropriate κ-point mesh was selected based on the Monkhorst–Pack scheme. The self-consistent of first-principles calculations were considered converged when the maximum force on the atom, maximum displacement between cycles, maximum stress, and total energy changes were less than 0.01 eV/Å, 5.0 × 10^−4^ Å, 0.02 GPa, and 5.0 × 10^−6^ eV/atom, respectively.

### Evaluation of apoptosis induced by Ti_3_C_2_

The study investigated the apoptosis of 4T1 cells using the Annexin V/PI apoptosis kit. The cells were seeded in a 6-well plate at a density of 10^6^ cells per well. After 24 h, Ti_3_C_2_ was added to the medium and incubated for 8 h. Following laser irradiation, the cells were harvested and stained with the Annexin V/PI apoptosis kit for FCM analysis.

### Western blotting analysis

The cells were lysed, and total protein was extracted. Western blotting was performed as previously described. Protein concentration was determined using a BCA Protein Assay Kit (P0012S, Beyotime, Shanghai, China). Protein samples (20 μg/lane) were separated on a 12.5% sodium dodecyl sulfate polyacrylamide gel by electrophoresis and transferred to a polyvinylidene fluoride membrane. The membrane was blocked with 5% nonfat milk at room temperature for 1.5 h. Primary antibodies Anti-GPX4 (1:1000, 52455S, Cell Signaling Technology, MA, USA) and glyceraldehyde 3-phosphate dehydrogenase monoclonal antibody-horseradish peroxidase (1:20,000, MB001H, Bioworlde, MN, USA) were incubated overnight at 4 °C. After washing the membrane 3 times with tris-buffered saline with Tween 20 for 30 min, a secondary antibody (S0001, 1:5000, Affinity, Jiangsu, China) was added and incubated. Protein bands were detected using an ultrasensitive electrochemiluminescence kit (Jingxin, Guangzhou, China) and analyzed with ImageJ 6.0 software (National Institutes of Health, Bethesda, Maryland, USA) through an imaging system (iBright CL750, Invitrogen, Hercules, MA, USA).

### The anticancer effect of Ti_3_C_2_ in vivo

The Animal Ethic Committee of Fifth Affiliated Hospital of Sun Yat-sen University approved the in vivo antitumor effect of Ti_3_C_2_. Balb/c mice were subcutaneously injected with 4T1 cancer cells on their back. After a week, the mice were treated with one of the following: PBS, Ti_3_C_2_, laser, and Ti_3_C_2_ plus laser (1.5 W/cm^2^, 5 min). Tumor volume and body weight were measured every 4 d. After the fifth treatment, the mice were anesthetized and main organs and tumors were harvested and fixed in paraffin for further immunohistochemical assays. Routine blood tests were conducted to assess peripheral blood.

### Statistical analysis

The experiments were conducted 3 times, and all comparisons were analyzed using either the Student *t* test or 2-way analysis of variance (ANOVA) with GraphPad 9.0. Statistical significance was marked as follows: ns for *P* > 0.05; * for *P* < 0.05;** for *P* < 0.01; *** for *P* < 0.001; and **** for *P* < 0.0001.

## Results

### Ti_3_C_2_ nanozyme preparation and characterization

**Fig. 2.**
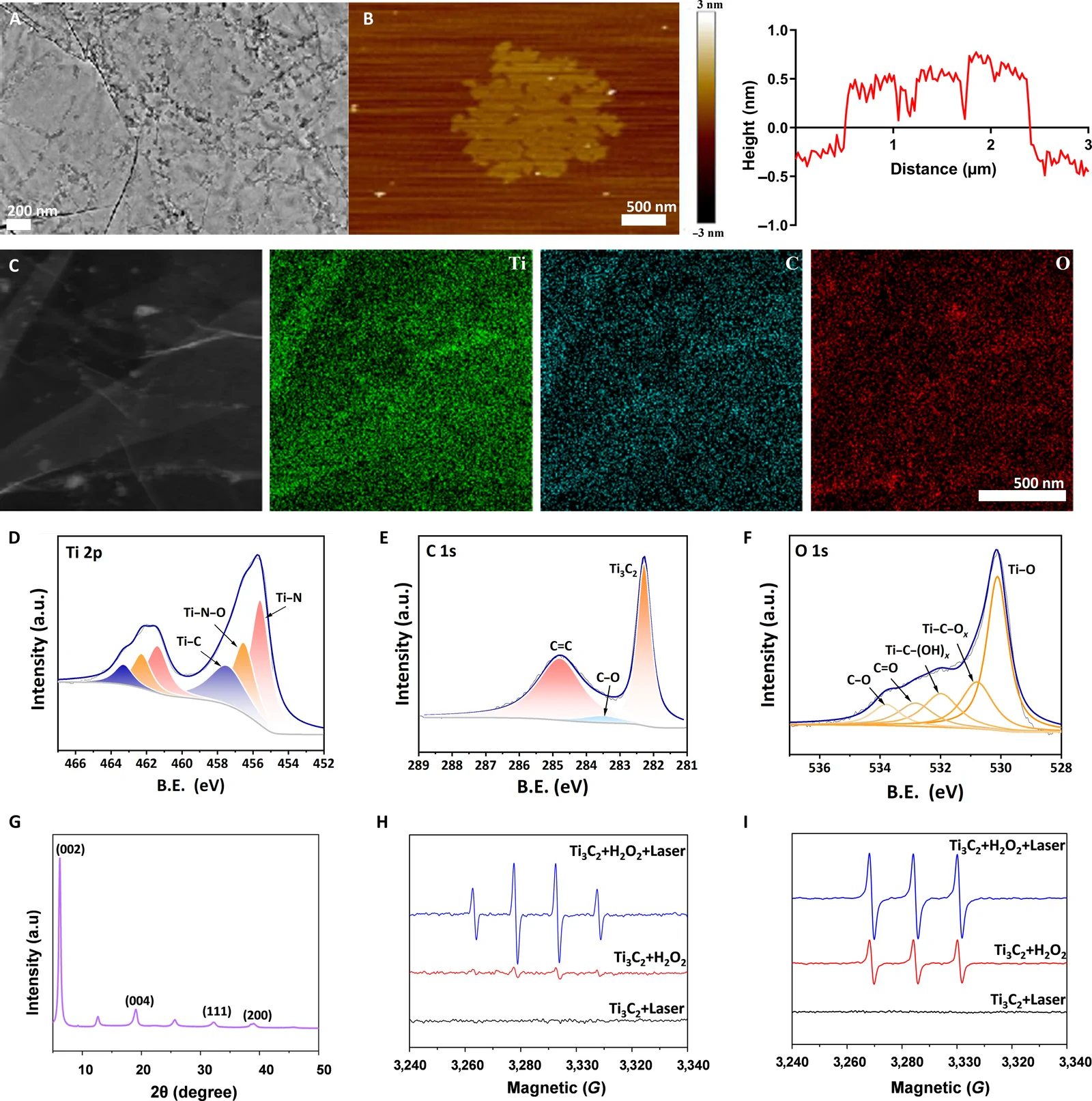
Characterization of Ti_3_C_2_ nanosheets. (A) The transmission electron microscopy (TEM) image of Ti_3_C_2_ nanosheets. The scale bar is 200 nm. (B) The atomic force microscopy (AFM) image of Ti_3_C_2_ nanosheets. The scale bar is 500 nm. (C) The transmission electron microscopy (TEM) image of Ti_3_C_2_ nanosheets and corresponding energy-dispersive x-ray spectroscopy (EDX) mapping. The scale bar is 500 nm. (D) The Ti2p spectrum of Ti_3_C_2_ nanosheets. (E) The C1s spectrum of Ti_3_C_2_ nanosheets_._ (F) The O1s spectrum of Ti_3_C_2_ nanosheets. (G) X-ray diffraction (XRD) pattern of Ti_3_C_2_ nanosheets. (H) The electron spin resonance (ESR) spectrum for detecting ·OH using 5,5-dimethyl-1-pyrroline N-oxide (DMPO). (I) The ESR spectrum for detection of ^1^O_2_ using 2,2,6,6-tetramethylpiperidine hydrochloride (TEMP).

The Ti_3_C_2_ nanozyme was produced via chemical exfoliation. A mixture of lithium fluoride and hydrochloric acid was used to remove the surface oxide layer, and ultrathin Ti_3_C_2_ nanosheets were obtained using selective etching techniques. The obtained Ti_3_C_2_ exhibits a 2D structure, as shown in Fig. 2A and B. EDS analysis revealed the presence of Ti, C, and O elements within Ti_3_C_2_, as illustrated in Fig. 2C. XPS measurements were performed to investigate the surface chemical compositions of Ti_3_C_2_ nanosheets. The high-resolution spectrum of Ti2p exhibited prominent peaks at 457.56 eV, corresponding to Ti2p3/2 (Fig. 2D). The asymmetric C1s spectrum was deconvoluted into 3 peaks at 287.36, 284.81, and 283.56 eV, which correspond to C=C, C–O, and Ti–C, respectively (Fig. 2E). Deconvolution of the O1s XPS spectrum (Fig. 2F) reveals the following chemical bonds: Ti–O (530.01 eV), Ti–(OH)*_x_* (532.01 eV), and Ti–C–O*_x_* (533.8 eV). The alteration in the valence state of Ti_3_C_2_ upon reaction with H_2_O_2_ was also examined using XPS, as depicted in Fig. S1. The Ti2p spectrum clearly showed the presence of Ti^4+^, indicating the formation of Ti^4+^ resulting from the reaction between Ti_3_C_2_ and H_2_O_2_. Subsequently, further interaction between Ti^4+^ and H_2_O_2_ led to the generation of an orange-colored peroxo-titanic acid [17]. Moreover, we employed an x-ray powder diffractometer to study the crystal structure of Ti_3_C_2_ nanosheets, revealing diffraction peaks that exhibited resemblance to those previously reported in the literature for Ti_3_C_2_ nanosheets (Fig. 2G) [18].

### Multienzyme activity of Ti_3_C_2_ nanosheets

**Fig. 3.**
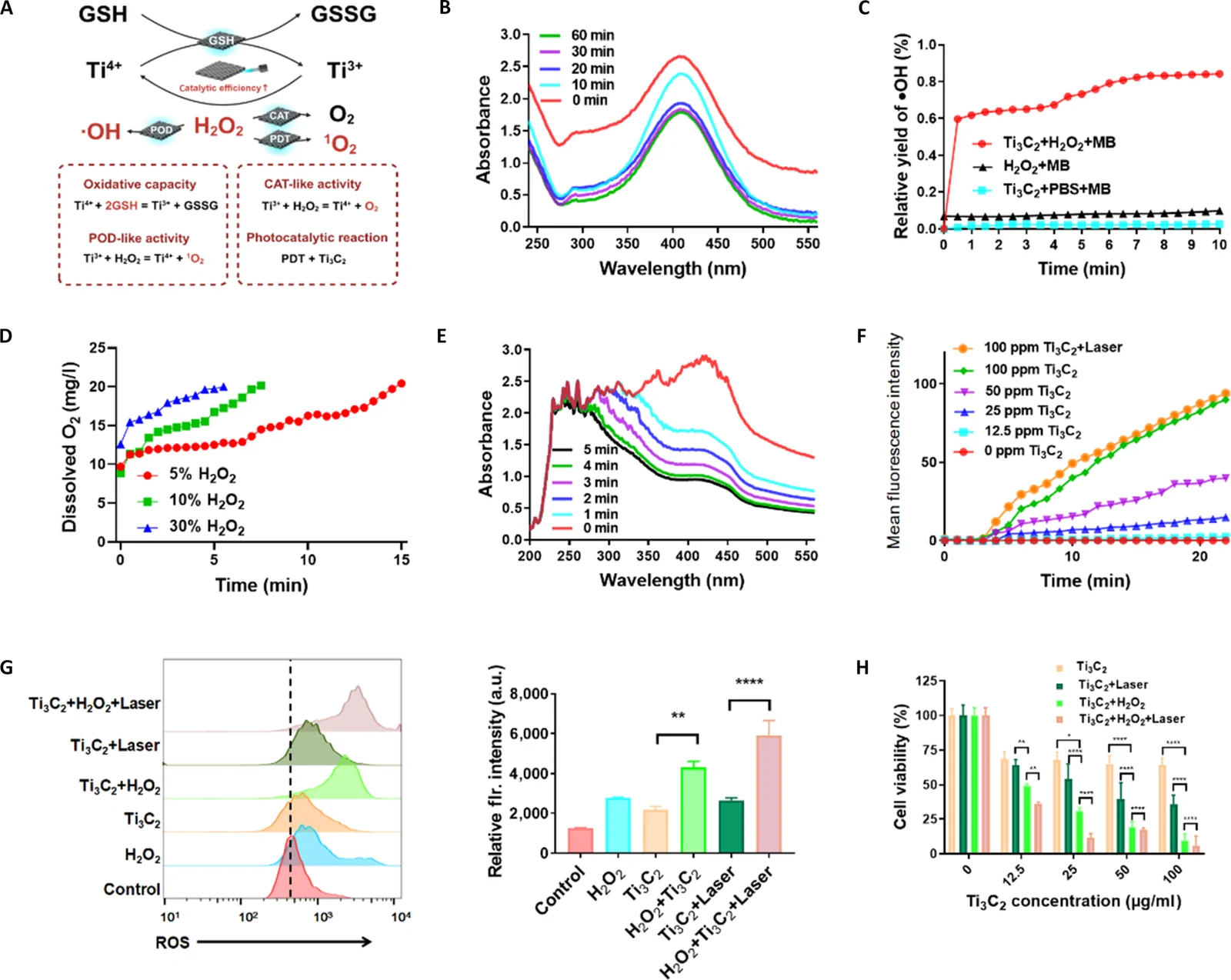
The multienzyme mimicking activity of Ti_3_C_2_ nanosheets. (A) Ti_3_C_2_ nanosheets multienzyme activity scheme. (B) Glutathione peroxidase (GPX)-mimicking activity of Ti_3_C_2_ nanosheets using 5,5′-dithiobis-2-nitrobenzoic acid (DTNB) assay. (C) The generation of ·OH by Ti_3_C_2_ nanosheets reaction with H_2_O_2_. (D) The generation of O_2_ by Ti_3_C_2_ nanosheets reacted with H_2_O_2_. (E) Time-dependent absorption spectra of 1,3-diphenylisobenzofuran (DPBF) treated by Ti_3_C_2_ under 1,064-nm irradiation (1.0 W/cm^2^). (F) The reactive oxygen species (ROS) generation of different concentrations of Ti_3_C_2_ nanosheets was incubated with 4T1 cells. (G) ROS generation of 4T1 cells treated by Ti_3_C_2_ nanosheets and laser irradiation. (H) In vitro antitumor effect of Ti_3_C_2_ nanosheets.

Nanozymes possessing enzyme-mimicking capabilities exhibit properties that are potentially advantageous for tumor therapy [10]. The catalytic activity was assessed via electron spin resonance spectroscopy by detecting the generation of ·OH and ^1^O_2_, serving as indicators (Fig. 2H and I). As shown in Fig. 3A, the POD-like activity catalyzes H_2_O_2_ decomposition into ·OH, while the CAT-like activity converted H_2_O_2_ into O_2_, alleviating tumor hypoxia [19]. GSH, a pivotal redox buffer, plays a crucial role in tumor progression [20]. Elevated GSH levels are indispensable for effectively scavenging excessive ROS; hence, GSH depletion can enhance ROS-based antitumor therapy [21]. The GPX-like activity of Ti_3_C_2_ nanosheets was determined using 5,5′-dithiobis-2-nitrobenzoic acid. As illustrated in Fig. 3B, Ti_3_C_2_ nanosheets effectively consumed the GSH solution in a time-dependent manner, as evidenced by a decreased absorption peak at 412 nm of sulfhydryl groups. Ti_3_C_2_ nanosheets demonstrated dual enzyme-mimicking activities under physiological conditions (Fig. 3C and D). The catalytic ability of Ti_3_C_2_ in generating ^1^O_2_ was further investigated using the 1,3-diphenylisobenzofuran probe (Fig. 3E). A time-dependent increase in ^1^O_2_ generation through Ti_3_C_2_ catalytic activity was enhanced by laser irradiation.

Real-time ROS generation, detected by the Incucyte S3 system using DCFH-DA probe, revealed a time-dependent increase in ROS accumulation over time in cancer cells induced by Ti_3_C_2_ (Fig. 3F and Fig. S2). The generation of ROS was further enhanced by NIR-II laser irradiation. Figure 3G illustrates the examination of ROS production in the Fenton-like reaction using a DCFH-DA probe in TNBC cells. The results demonstrated a marked increase in ROS levels within cancer cells upon H_2_O_2_ and laser irradiation, suggesting that Ti_3_C_2_ nanosheets effectively elevate intracellular ROS. Notably, the Ti_3_C_2_+H_2_O_2_+Laser group exhibited the highest ROS level, suggesting that laser irradiation effectively enhanced the catalytic activity of Ti_3_C_2_ nanosheets. The cytotoxicity of the Ti_3_C_2_-catalyzed reaction was evaluated via the CCK8 assay. As shown in Fig. 3H, the Ti_3_C_2_+Laser group exhibited higher cytotoxicity, while the Ti_3_C_2_+H_2_O_2_+Laser showed the lowest cell viability. The results demonstrated a dose-dependent decrease in cell viability induced by Ti_3_C_2_, with both laser irradiation and H_2_O_2_ potentiating its antitumor effect. The Ti_3_C_2_ shows negligible cytotoxicity to normal cells (Fig. S3).

### The study of catalytic activity by DFT calculations

**Fig. 4.**
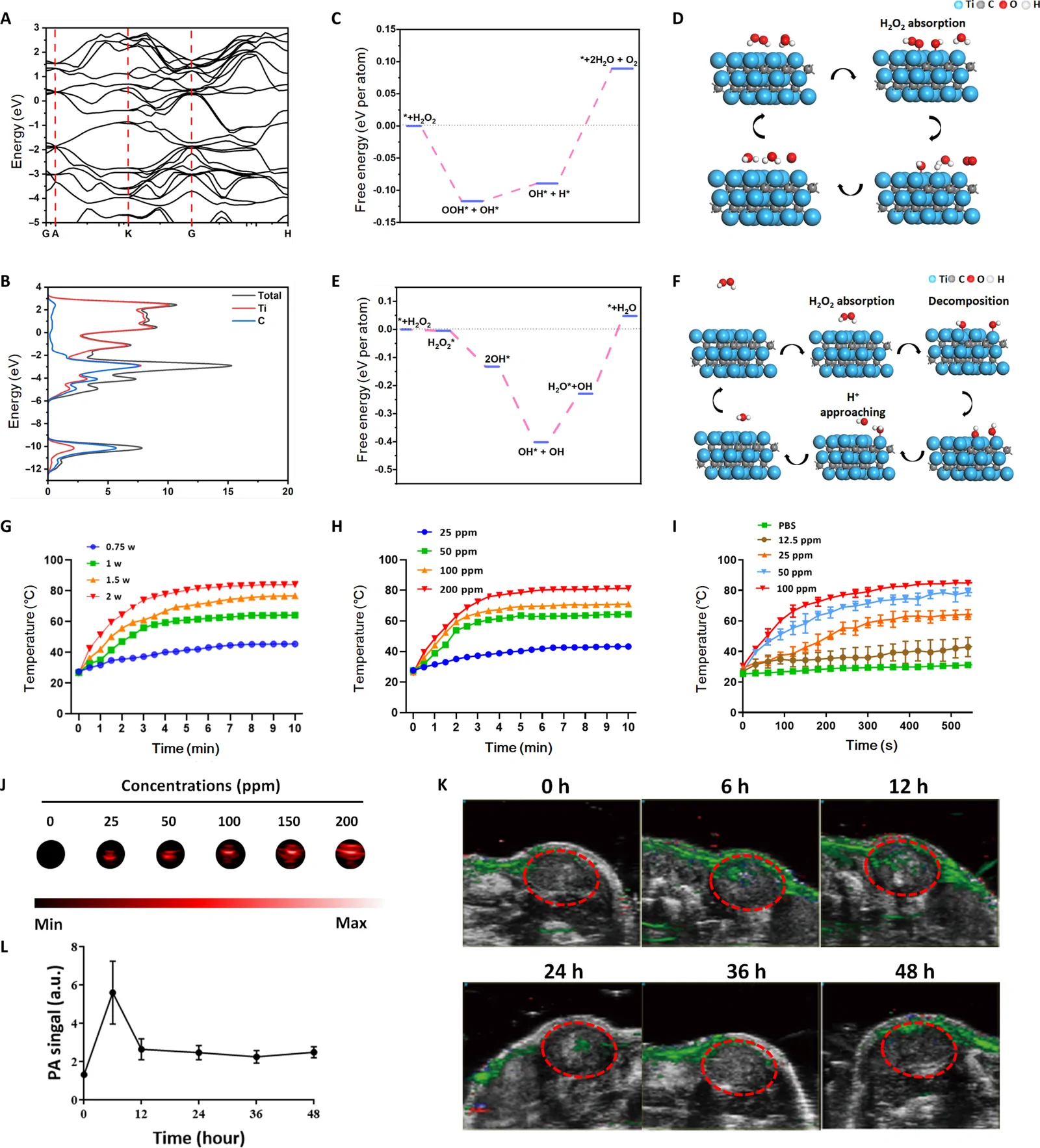
The density functional theory (DFT) explorations, photothermal, and photoacoustic (PA) properties of Ti_3_C_2_ nanosheets. (A) Band structure of Ti_3_C_2_ and (B) the corresponding electron density of states projected onto the C and Ti atoms. (C and D) The free energy diagrams and corresponding reaction pathways of catalase (CAT)-like activity on the (001) surface of Ti_3_C_2_. (E and F) The free energy diagrams and corresponding reaction pathways of peroxidase (POD)-like activity on the (001) surface of Ti_3_C_2_. (G) Photothermal conversion of Ti_3_C_2_ nanosheets at different power densities. (H) Photothermal conversion of Ti_3_C_2_ nanosheets at different concentrations. (I) The concentration-dependent photothermal heating curves of Ti_3_C_2_ nanosheets incubated with 4T1 cells. (J) Photoacoustic images of Ti_3_C_2_ nanosheets at different concentrations. (K) The photoacoustic images and (L) quantities of Ti_3_C_2_ in vivo.

To investigate Ti_3_C_2_’s catalytic activity, we conducted first-principles calculations using the Cambridge Serial Total Energy Package code based on DFT. Initially, we optimized the crystal structures of the (001) surface of Ti_3_C_2_ employing the Broyden–Fletcher–Goldfarb–Shanno method, as depicted in Fig. S4. Band structure analysis (Fig. 4A) demonstrates Ti_3_C_2_’s metallic characteristics due to the absence of a significant band gap. Examination of the density of states highlights the primary contribution of *d*-orbital electrons of Ti to the total energy level at the Fermi surface (at 0 eV on the abscissa), with a smaller contribution from the *p*-orbitals of C (Fig. 4B). Then, we calculated the equilibrium crystal structures of the (001) surface of Ti_3_C_2_ and the adsorbed H_2_O_2_ surface using the Broyden–Fletcher–Goldfarb–Shanno optimization method. Subsequent calculations of adsorption energies for intermediates during H_2_O_2_ decomposition reveal their relative value to the adsorbed H_2_O_2_ structure. The free energy diagrams in Fig. 4C and E reveal that intermediates display negative adsorption energies, indicating stable adsorption during a hydroxyl radical desorption process.

Based on the foregoing analysis, we propose the presence of CAT-like activity on the (001) surface of Ti_3_C_2_. The energy distribution and corresponding reaction pathways are shown in Fig. 4C and D. According to the proposed mechanism, 2 H_2_O_2_ molecules are initially adsorbed onto the Ti_3_C_2_ surface, decomposing to form OOH and OH adsorbed on the outer Ti atoms. The energy loss for the generation of H_2_O molecules from the remaining H and OH is 0.117 eV, indicating that the reaction is thermodynamically feasible. Subsequently, OOH* and OH* adsorbed on Ti atoms react to form OH and H, respectively, while releasing O_2_. This step is found to be the rate-determining step, as it results in an energy gain of 0.028 eV. Finally, Ti_3_C_2_ exhibits a relatively weak binding affinity for H_2_O, resulting in an energy gain of 0.089 eV when OH* and H* combine to form the H_2_O molecule. These findings corroborate the hypothesis that Ti_3_C_2_ mimics the CAT-like catalytic activity, which facilitates the conversion of H_2_O_2_ into O_2_.

Moreover, the reaction pathways and energy distribution of POD-like activity are illustrated in Fig. 4E and F. The results demonstrated that H_2_O_2_ was captured by Ti_3_C_2_ and adsorbed onto Ti atoms with adsorption energy of −0.004 eV, indicating favorable energetics for H_2_O_2_ adsorbed onto Ti_3_C_2_. Subsequently, the reaction proceeds to the decomposition of the intermediate into 2 OH* ions, accompanied by a reduction in energy of 0.269 eV. Concurrently, the remaining OH* undergoes a reaction with the approaching H^+^ to form H_2_O adsorbed on the surface. This reaction demonstrates the thermodynamically favorable conversion of OH* into H_2_O. Overall, the multienzymatic-mimicking activity of Ti_3_C_2_ has been theoretically validated by a detailed investigation of the proposed reaction pathways and energy distributions.

### The photothermal and photoacoustic property of Ti_3_C_2_ nanosheets

The multienzyme mimicking activities of Ti_3_C_2_ enable efficient generation of ROS upon external laser stimulation, rendering it a promising candidate for tumor treatment. Moreover, Ti_3_C_2_ exhibits superior NIR laser absorption capability, facilitating effective conversion of light energy to heat. With increasing laser power from 0.75 to 2.0 W/cm^2^, the temperature of Ti_3_C_2_ rose from 30 to 80 °C. Additionally, a correlation between the concentration of Ti_3_C_2_ and the temperature of the solution was observed (Fig. 4G and H). These results suggest that Ti_3_C_2_ exhibits excellent photothermal performance and exceptional stability. The temperature of the Ti_3_C_2_ within tumor cells rapidly escalated beyond 80 °C with prolonged irradiation time. In contrast, cells incubated with PBS showed no significant temperature increase. These results highlight the exceptional photothermal conversion efficiency exhibited by Ti_3_C_2_ nanosheets. Photoacoustic (PA) imaging, a noninvasive technique that combines ultrasound and optical imaging modalities, provides enhanced spatial resolution and increased imaging depth compared to traditional optical imaging methods, enabling in vivo tissue penetration of up to 70 mm [22]. Due to its strong NIR absorption and excellent photothermal conversion capabilities, Ti_3_C_2_ demonstrates suitability for in vivo PA imaging investigation. Figure 4J illustrates the concentration-dependent increase in PA signal intensity of Ti_3_C_2_. Notably, Ti_3_C_2_ exhibits remarkable performance in PA imaging by efficiently accumulating at tumor sites. The effective accumulation of Ti_3_C_2_ in the tumor was confirmed through in vivo experiments (Fig. 4K and L). This unique capability enables its utilization for imaging guidance, laser amplification, and efficient enzyme-catalyzed therapeutic performance.

### Ti_3_C_2_ nanosheets induce mitochondrial damage in TNBC cells

**Fig. 5.**
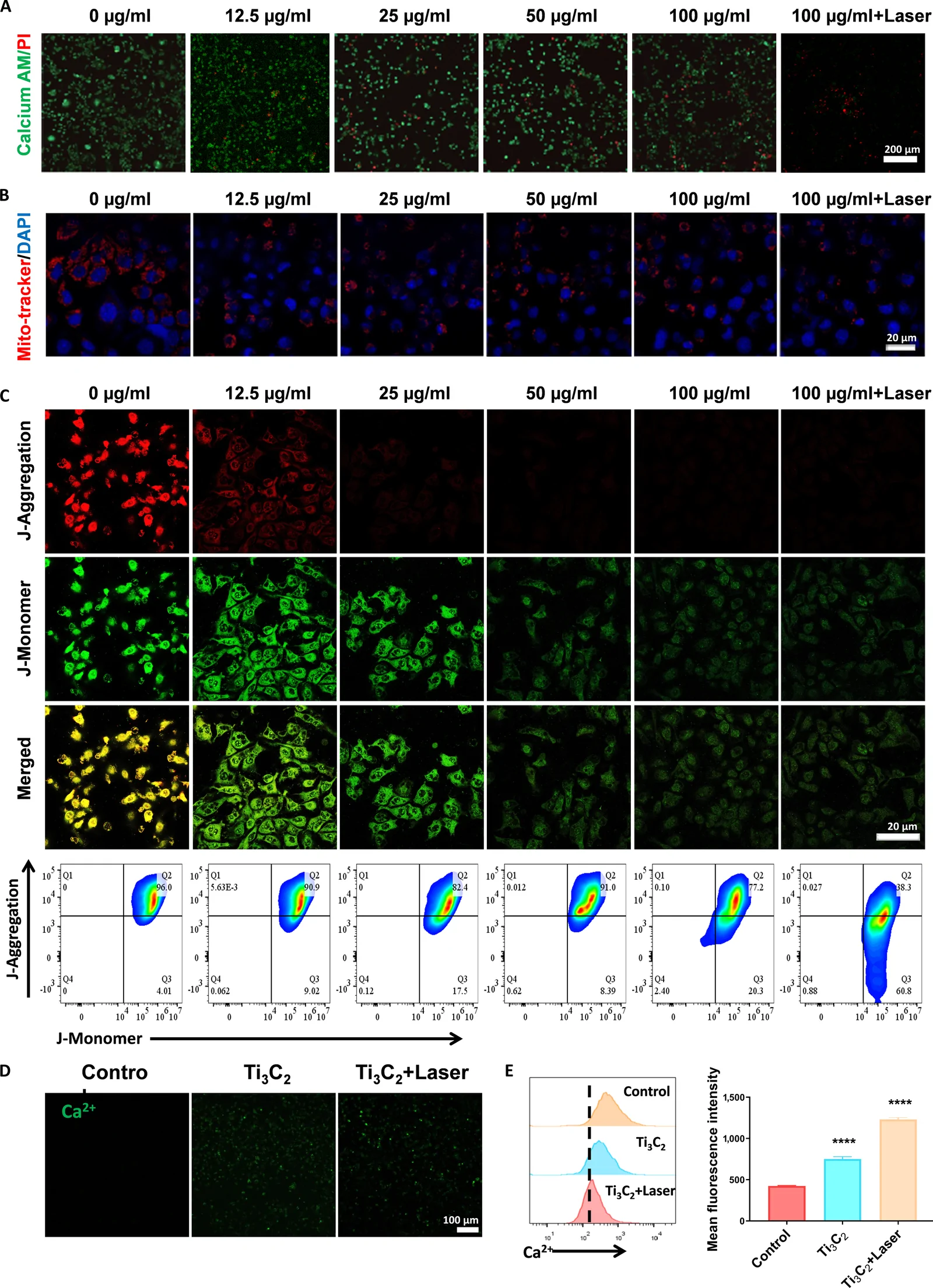
The calcium overload induced by Ti_3_C_2_’s catalytic activity. (A) Live/dead double staining of 4T1 cells with different treatments indicated by Calcein-AM (green, live cells) and propidium iodide (PI) (red, dead cells). Scale bar: 200 μm. (B) Confocal laser scanning microscopy (CLSM) images of mitochondria damage of 4T1 cells with different treatments. Scale bar: 20 μm. (C) The mitochondria damage of 4T1 cells with different treatments. Scale bar: 20 μm. (D) CLSM images of Ca^2+^ in 4T1 cells with different treatments. Scale bar: 100 μm. (E) Flow cytometry (FCM) and quantification results of Ca^2+^ in 4T1 cells with different treatments.

The ferroptosis-like therapy capability of Ti_3_C_2_ nanosheets was observed through live/dead cell staining using calcein AM/PI assay (Fig. 5A). The concentration of Ti_3_C_2_ nanosheets exhibited a positive ​correlation with the intensity of red fluorescence signal, which served as an indicator for cell death. The exceptional photothermal conversion synergized with enzymatic activity to induce cell death. Mitochondrial damage was further investigated using the Mito-tracker probe, demonstrating a direct correlation between Ti_3_C_2_ concentration and the extent of the mitochondrial damage inflicted on tumor cells. As shown in Fig. 5B, the Ti_3_C_2_-based catalytic treatments induced significant mitochondrial impairment due to the catalytic activity of nanozymes. Moreover, JC-1 staining results showed the multienzyme activity with photothermal therapy resulted in the most severe mitochondrial damage (Fig. 5C).

Excessive calcium influx can result in mitochondrial damage and subsequent of apoptosis of cancer cells. Disruption of calcium homeostasis within tumor cells can be induced by ROS [23]. Considering the robust ROS generation and the mitochondrial damage induced by the multienzyme mimicking activity of Ti_3_C_2_, it can be postulated that Ti_3_C_2_ may mediate Ca^2+^ overload. The Fluo-4 AM fluorescence probe was utilized to investigate the intracellular Ca^2+^ levels in cancer cells, indicating that Ti_3_C_2_ nanosheets induced an accumulation of Ca^2+^ in cancer cells, which was further augmented by laser irradiation (Fig. 5D and E).

### The ferroptosis and the immunomodulatory effects induced by Ti_3_C_2_ nanosheets

**Fig. 6.**
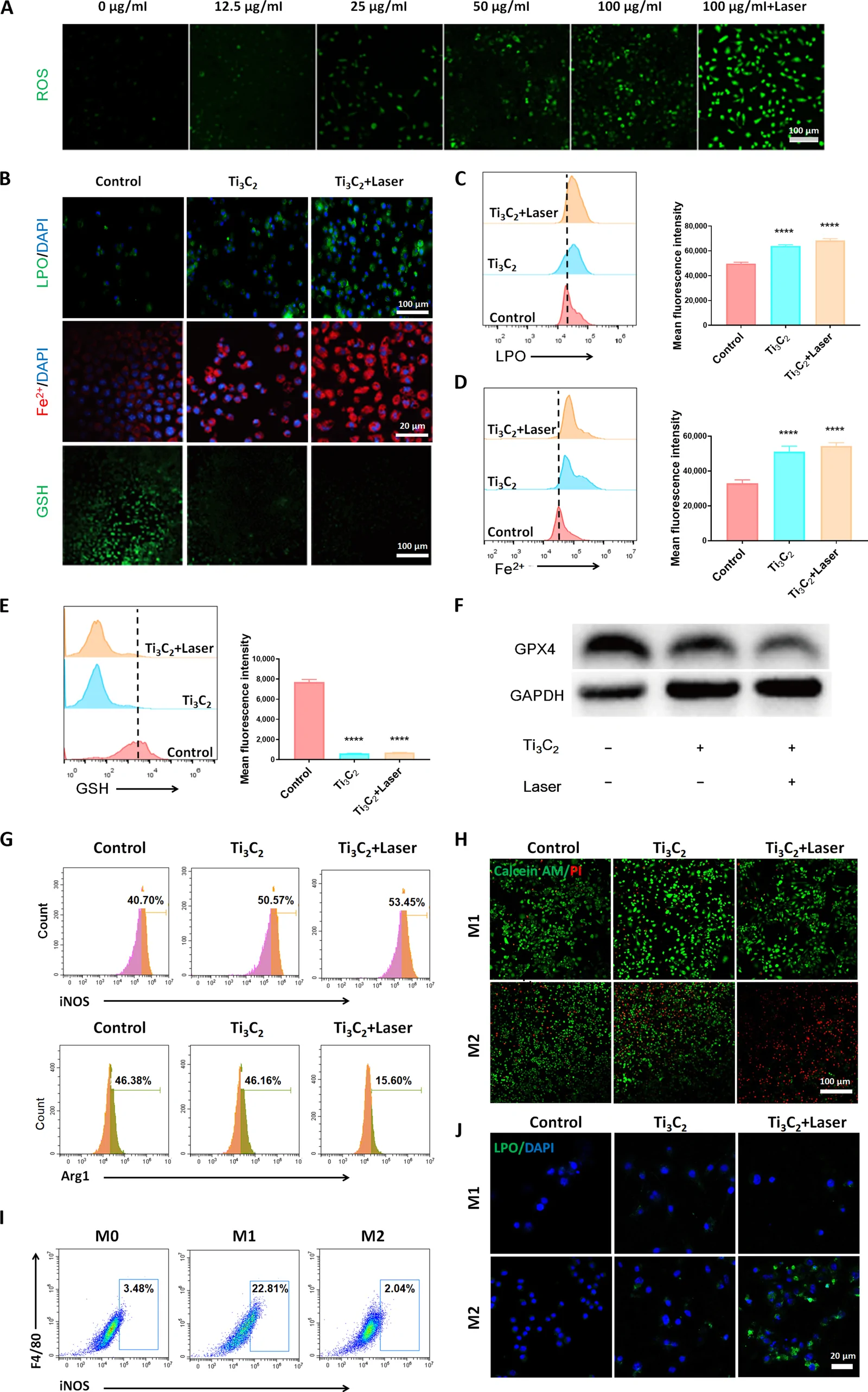
The ferroptosis and immunomodulation induced by the catalytic activity of Ti_3_C_2_. (A) Confocal laser scanning microscopy (CLSM) images of reactive oxygen species (ROS) in 4T1 cells with different treatments. Scale bar: 100 μm. (B) CLSM images of glutathione (GSH), Fe^2+^, and lipid peroxidation (LPO) in 4T1 cells with different treatments. Scale bar: 100 μm (top), 20 μm (middle), and 100 μm (bottom). (C) Flow cytometry (FCM) results of LPO in 4T1 cells with different treatments. (D) FCM results of Fe^2+^ in 4T1 cells with different treatments. (E) FCM results of GSH in 4T1 cells with different treatments. (F) The expression of glutathione peroxidase 4 (GPX4) in 4T1 cells with different treatments. (G) The inducible nitric oxide synthase (iNOS) and Arg1 expression in macrophages incubated with different cell supernatants. (H) CLSM images of live/dead M1 and M2 macrophages after different treatments. Scale bar: 100 μm. (I) FCM analysis of iNOS expression in M1 and M2 macrophages. (J) CLSM images of LPO expression in M1 and M2 macrophages. Scale bar: 20 μm.

Ferroptosis, a regulated form of cell death characterized by elevated ROS production and lipid peroxidation, leads to cellular demise [5]. As shown in Fig. 6A and B, Ti_3_C_2_ nanosheets induce elevated ROS production and lipid peroxidation in cancer cells, which is further exacerbated by laser irradiation. This elevation in lipid peroxidation is accompanied by a depletion of GSH, which is an important antioxidant [24]. These events are critical in the process of ferroptosis and play a crucial role in maintaining cellular redox homeostasis [25]. In Fig. 6B, the FerroOrange probe was used to detect the levels of Fe^2+^ in cancer cells following various treatments, demonstrating that Ti_3_C_2_ treatment induces intracellular accumulation of Fe^2+^, aligning with its role in promoting ferroptosis. The increased Fe^2+^ levels imply that Ti_3_C_2_ treatment triggers the ROS generation through the Fenton-like reaction, thereby contributing to lipid peroxidation and ultimately leading to ferroptosis (Fig. 6C to E). Moreover, this is supported by the significant decrease in the expression levels of GPX4, a characteristic enzyme involved in cellular ferroptosis (Fig. 6F). Together, these findings suggest that Ti_3_C_2_ nanosheets can initiate TME-responsive catalytic reactions, induce LPO damage, and ultimately trigger ferroptosis in cancer cells.

The presence of TAMs within the TME plays a pivotal role in facilitating tumor proliferation [26]. TAMs are classified into 2 main subtypes: M1 macrophages, which exhibit anticancer properties, and M2 macrophages, which promote tumorigenesis [27,28]. The polarization is often exacerbated by tumor hypoxia, which directs TAMs toward the M2 phenotype, consequently hindering the efficacy of immunotherapy [29]. Nevertheless, the M1-type TAMs possess the potential to enhance immune activation and elevate the concentration of intertumoral H_2_O_2_ [30], which in turn could improve the catalytic activity of nanozymes [31]. Therefore, the integration of nanozymes with CAT-like activity can overcome these conditions and alleviate the associated limitations [32]. Moreover, combining nanozyme-based ferroptosis-like therapy with TME immunomodulation holds great promise for enhancing the overall effectiveness of cancer treatment [33]. Ti_3_C_2_ reshaped the immunosuppressive TME by polarizing TAMs toward antitumor M1 phenotype (iNOS^+^, Arg1^−^) (Fig. 6G). FCM revealed M1 macrophages exhibited higher inducible nitric oxide synthase (iNOS) expression than M2, correlating with resistance to ferroptosis (Fig. 6I). Conversely, M2 macrophages showed higher cell death (PI^+^) and elevated LPO levels (Fig. 6H), indicating selective ferroptosis susceptibility. In contrast, M1 macrophages demonstrated resistance to ferroptosis. The accumulation of LPO in different macrophage subtypes has been demonstrated and illustrated in Fig. 6J. The integration of ferroptosis induction and M1 macrophage polarization achieved dual therapeutic effects: (a) direct tumor cell killing via LPO-driven ferroptosis, and (b) immune activation through TAM reprogramming.

### In vivo antitumor and the immune effect of Ti_3_C_2_ nanosheets

**Fig. 7.**
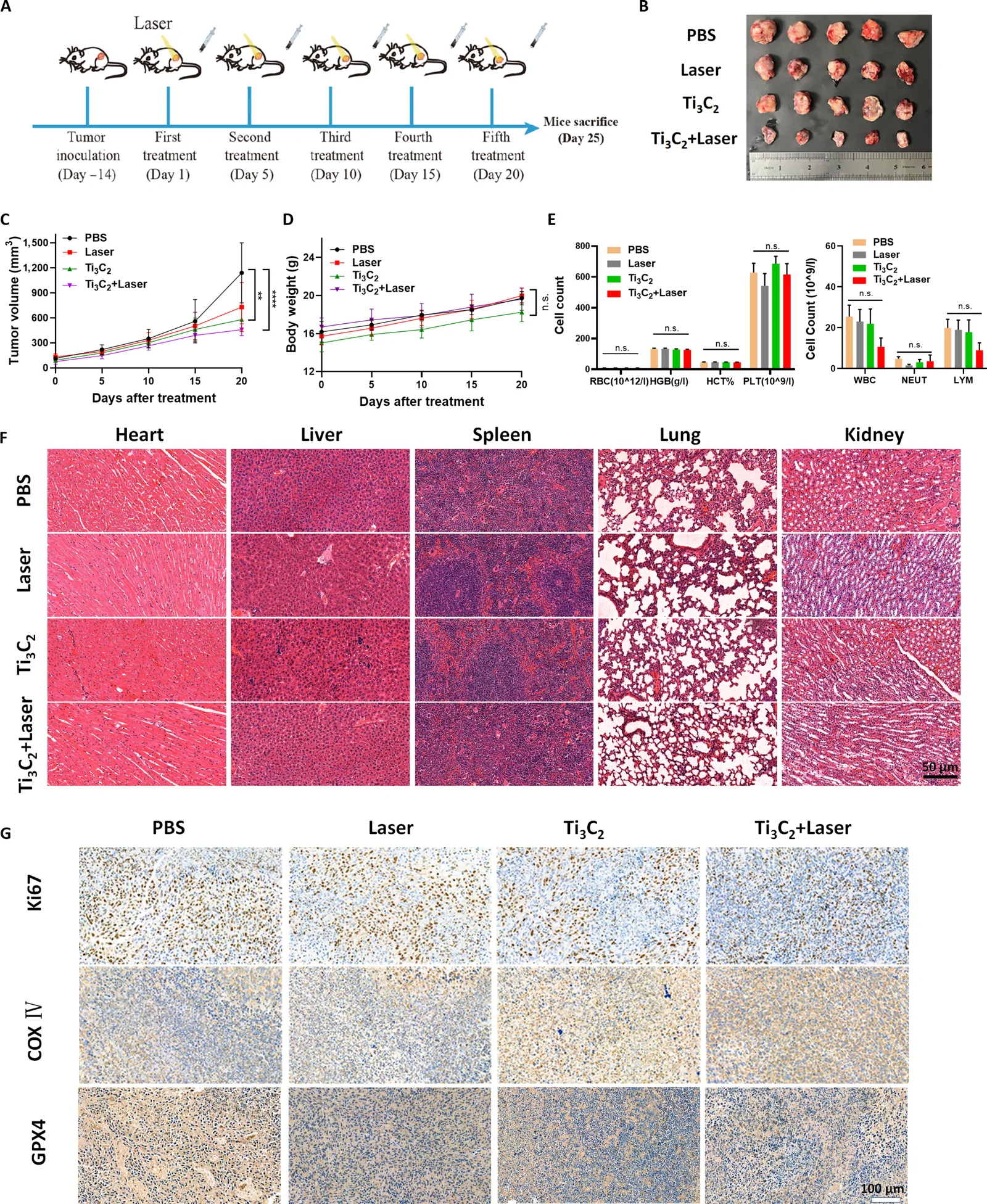
The antitumor and biocompatibility immunomodulation effect of Ti_3_C_2_ in vivo. (A) Schematic illustration of anticancer therapy in vivo. (B) The images of tumors after different treatments. (C) Tumor growth curves on tumor-bearing mice after various treatments. (D) Body weights of tumor-bearing mice after different treatments. (E) Blood routine of tumor-bearing mice after different treatments. (F) The hematoxylin and eosin (H&E) of the main organ in different treatments. Scale bar: 50 μm. (G) Expression of Ki67, COX IV, and glutathione peroxidase 4 (GPX4) were investigated by immunohistochemistry assays. Scale bar: 100 μm. n.s., not significant.

The in vivo antitumor effect was evaluated by creating a BALB/c mouse xenograft model of TNBC. The mice were randomly divided into 4 groups: PBS, laser, Ti_3_C_2_, and Ti_3_C_2_+laser. Ti_3_C_2_ (5 mg/kg) was administered via tail vein injection, followed by laser irradiation (1,064 nm, 1.5 W/cm^2^, 5 min) after 6 h, as shown in Fig. 7A. Subsequently, the mice were euthanized, and their main organs, including the heart, liver, spleen, lung, kidney, and tumor, were collected and fixed for further experimentation. Tumor volume in the Ti₃C₂+laser group was reduced by 60.58% compared to PBS controls, while laser-only treatment showed no significant inhibition (Fig. 7B and C). Importantly, the addition of laser irradiation to Ti_3_C_2_ treatment elicited the most significant inhibitory effect on the tumor, suggesting a synergistic interaction between Ti_3_C_2_ and laser treatment in suppressing tumor growth. The body weight of the mice in the different treated groups did not exhibit any significant differences, indicating the safety profile of the treatment (Fig. 7D). To investigate the biocompatibility of the treatments, routine blood tests were conducted. The results indicated no significant differences between the white blood cells, neutrophil count, and red blood cells, as depicted in Fig. 7E. Histological analysis of major organs using hematoxylin and eosin staining under different treatment conditions is presented in Fig. 7F. Notably, Ti_3_C_2_ nanozyme-mediated immunomodulation-enhanced catalytic tumor therapy exhibited remarkable biocompatibility. The immunohistochemistry demonstrated that Ti_3_C_2_+laser treatment down-regulated Ki67 and GPX4, while up-regulating COX IV (Fig. 7G).

**Fig. 8.**
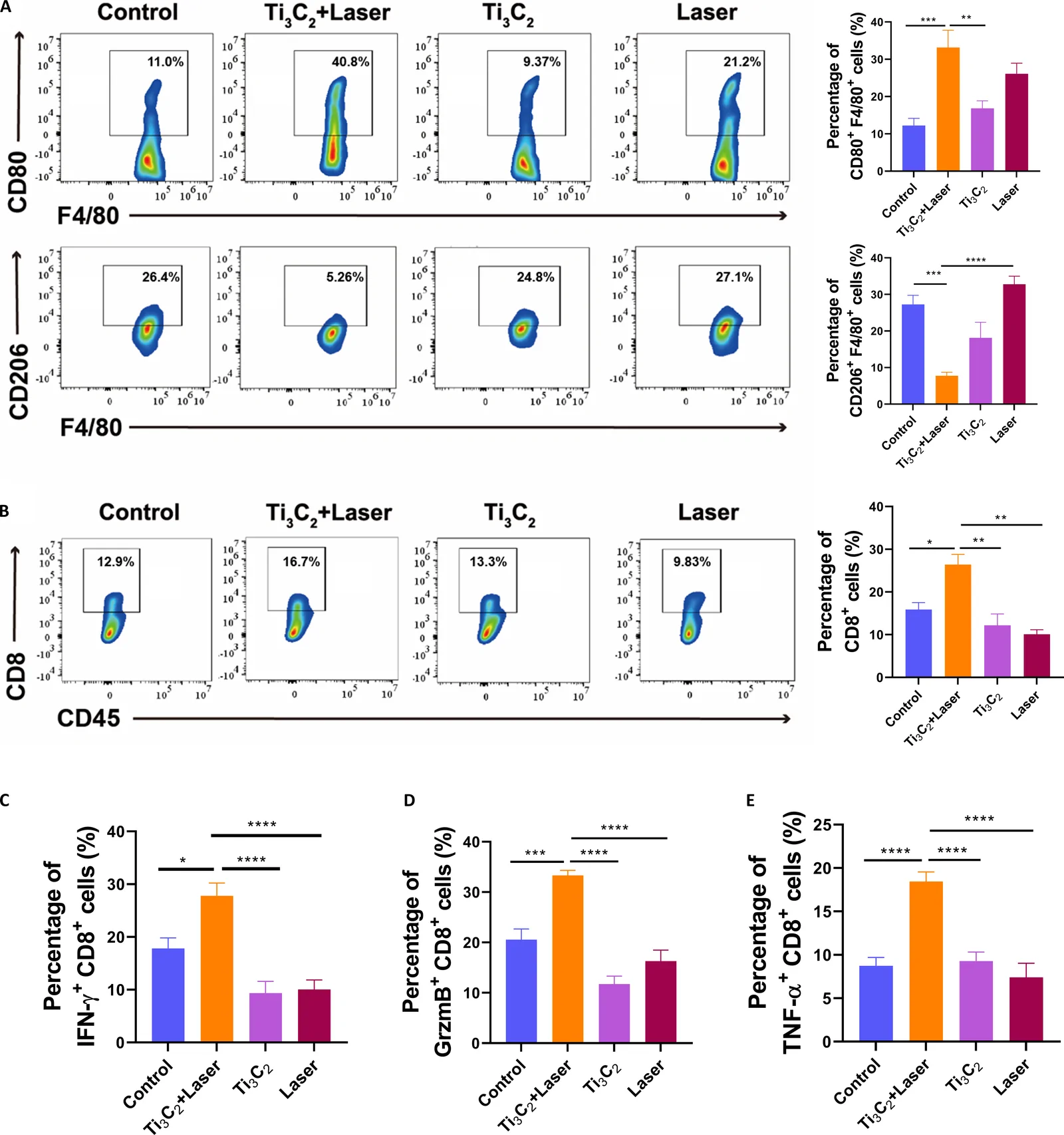
The immunomodulation effect of Ti_3_C_2_ in vivo. (A) Flow cytometry results of tumor-associated macrophages (TAMs) from tumor tissues. (B) The CD8^+^ T cells from tumor tissues treated by different treatments. (C) The IFN-γ^+^CD8^+^ T cells from tumor tissues treated by different treatments. (D) The GzmB^+^CD8^+^ T cells from tumor tissues treated by different treatments. (E) The TNF-α^+^CD8^+^ T cells from tumor tissues treated by different treatments.

In this study, we aimed to investigate the immunomodulatory effect of Ti_3_C_2_ by analyzing tumor and spleen cells using FCM. Ti_3_C_2_ reprogrammed TAMs toward the antitumor M1 phenotype (CD80^+^), increasing their proportion from 11% (PBS group) to 40.8% (Ti_3_C_2_+laser group) (Fig. 8A and Fig. S5). CD8^+^ T cells recognize and eliminate infected cells or cancerous cells through the recognition of specific antigens [34]. In our study, we observed a significant increase in percentage of CD8^+^ T cells following treatment with Ti_3_C_2_ and laser therapy, suggesting enhanced activation and recruitment of CD8^+^ T cells to improve antitumor immune response (Fig. 8B). Treatment of the TME with Ti_3_C_2_ could induce polarization of the macrophages into M1-type macrophages, augmenting their phagocytic activity and synergizing with Ti_3_C_2_’s tumor cell-killing effect to inhibit tumor proliferation. Furthermore, Ti_3_C_2_-induced ferroptosis elicits the production of tumor-specific antigens, thereby activating systemic T-cell immune responses that secrete cytotoxic factors such as tumor necrosis factor-α, Granzyme B (GzmB), and interferon γ. Consequently, this amplifies the killing capacity of CD8^+^ T cells against tumors (Fig. 8C). This dual mechanism suggests that Ti_3_C_2_ treatment can not only directly eliminate tumor cells but also stimulate an immune response against the tumor. Overall, our findings indicate that combining Ti_3_C_2_ with photothermal therapy enhances CD8^+^ T cells activation, promotes macrophages polarization toward an antitumor phenotype, and generates tumor-specific antigens to facilitate an improved antitumor immune response.

## Conclusions

This study presents a novel ferroptosis-driven therapeutic strategy for TNBC utilizing 2D Ti_3_C_2_ nanosheets, which integrate nanocatalytic activity, photothermal effects, and immunomodulation synergistically. The Ti_3_C_2_-based approach has been demonstrated to exhibit dual enzyme-mimetic acidity. The decomposition of H_2_O_2_, a by-product of tumor activity, into highly levels of ROS is a hallmark of the Ti_3_C_2_-based approach. Additionally, it exhibits CAT-like activity, converting H_2_O_2_ into O_2_, thereby alleviating tumor hypoxia. It is worth noting that the ferroptosis process, characterized by GSH depletion and GPX4 down-regulation, disrupts redox homeostasis, leading to lipid peroxidation and mitochondrial dysfunction. This Ti_3_C_2_-based platform overcomes the limitations of conventional ferroptosis inducers by combining spatiotemporal control, immune activation, and hypoxia alleviation, offering a transformative strategy for TNBC therapy.

## Data Availability

The data that support the findings of this study are available from the corresponding authors upon reasonable request.

## References

[1] Bianchini G, De Angelis C, Licata L, Gianni L. Treatment landscape of triple-negative breast cancer — Expanded options, evolving needs. Nat Rev Clin Oncol. 2022;19(2):91–113. 10.1038/s41571-021-00565-2 34754128

[2] Sung H, Ferlay J, Siegel RL, Laversanne M, Soerjomataram I, Jemal A, Bray F. Global cancer statistics 2020: GLOBOCAN estimates of incidence and mortality worldwide for 36 cancers in 185 countries. CA Cancer J Clin. 2021;71(3):209–249. 10.3322/caac.21660 33538338

[3] Li J, He D, Li S, Xiao J, Zhu Z. Ferroptosis: The emerging player in remodeling triple-negative breast cancer. Front Immunol. 2023;14:1284057. 10.3389/fimmu.2023.1284057 PMC10623117 37928550

[4] Chen Y, Li W, Kwon S, Wang Y, Li Z, Hu Q. Small-molecule ferritin degrader as a pyroptosis inducer. J Am Chem Soc. 2023;145(17):9815–9824. 10.1021/jacs.3c01852 37094179

[5] Jiang X, Stockwell BR, Conrad M. Ferroptosis: Mechanisms, biology and role in disease. Nat Rev Mol Cell Biol. 2021;22(4):266–282. 10.1038/s41580-020-00324-8 PMC8142022 33495651

[6] Chen M, Tong X, Sun Y, Dong C, Li C, Wang C, Zhang M, Wen Y, Ye P, Li R, et al. A ferroptosis amplifier based on triple-enhanced lipid peroxides accumulation strategy for effective pancreatic cancer therapy. Biomaterials. 2024;309: Article 122574. 10.1016/j.biomaterials.2024.122574 38670032

[7] Zhou P, Zhang S, Wang M, Zhou J. The induction mechanism of ferroptosis, necroptosis, and pyroptosis in inflammatory bowel disease, colorectal cancer, and intestinal injury. Biomolecules. 2023;13(5):820. 10.3390/biom13050820 PMC10216135 37238692

[8] Zhang D, Wu C, Ba D, Wang N, Wang Y, Li X, Li Q, Zhao G. Ferroptosis contribute to neonicotinoid imidacloprid-evoked pyroptosis by activating the HMGB1-RAGE/TLR4-NF-kappaB signaling pathway. Ecotoxicol Environ Saf. 2023;253: Article 114655. 10.1016/j.ecoenv.2023.114655 36812867

[9] Yu H, Tang K, Cai Z, Lin X, Huang Y, Yu T, Zhang Q, Wang Q, Wu L, Yang L, et al. Carbon dots-based nanozyme for drug-resistant lung cancer therapy by encapsulated doxorubicin/siRNA cocktail. Int J Nanomedicine. 2023;18:933–948. 10.2147/IJN.S390984 PMC9960730 36852185

[10] Wang Q, Liu J, He L, Liu S, Yang P. Nanozyme: A rising star for cancer therapy. Nanoscale. 2023;15(30):12455–12463. 10.1039/d3nr01976d 37462391

[11] Zhou T-J, Wan X, Zhang M-M, Liu D-M, Huang L-L, Xing L, Wang Y, Jiang H-L. Tumor microenvironment-initiated lipid redox cycling for efficient triple-negative breast cancer therapy. Biomaterials. 2023;300: Article 122205. 10.1016/j.biomaterials.2023.122205 37348324

[12] Zhang S, Zhang Y, Feng Y, Wu J, Hu Y, Lin L, Xu C, Chen J, Tang Z, Tian H, et al. Biomineralized two-enzyme nanoparticles regulate tumor glycometabolism inducing tumor cell pyroptosis and robust antitumor immunotherapy. Adv Mater. 2022;34(50): Article e2206851. 10.1002/adma.202206851 36193764

[13] Mantovani A, Allavena P, Marchesi F, Garlanda C. Macrophages as tools and targets in cancer therapy. Nat Rev Drug Discov. 2022;21(11):799–820. 10.1038/s41573-022-00520-5 PMC9380983 35974096

[14] Xiang X, Wang J, Lu D, Xu X. Targeting tumor-associated macrophages to synergize tumor immunotherapy. Signal Transduct Target Ther. 2021;6(1):75. 10.1038/s41392-021-00484-9 PMC7900181 33619259

[15] Liu C, Yang P, Li J, Cao S, Shi J. NIR/pH-responsive chitosan hydrogels containing Ti3C2/AuNRs with NIR-triggered photothermal effect. Carbohydr Polym. 2022;295: Article 119853. 10.1016/j.carbpol.2022.119853 35988979

[16] Huang W-X, Li Z-P, Li D-D, Hu Z-H, Wu C, Lv K-L, Li Q. Ti3C2 MXene: Recent progress in its fundamentals, synthesis, and applications. Rare Metals. 2022;41(10):3268–3300.

[17] Li Z, Cao X, Ma C, Gao R, Sun T, Li C. Induced Ti^3+^ doping nano TiO_1.95_ for visible light photooxidation of Rhodamine B based on the peroxo titanic precursor. Mater Lett. 2016;184:123–126.

[18] Wu X, Chen T, Chen Y, Yang G. Modified Ti3C2 nanosheets as peroxidase mimetics for use in colorimetric detection and immunoassays. J Mater Chem B. 2020;8(13):2650–2659. 10.1039/d0tb00239a 32129422

[19] Chen Z, Han F, Du Y, Shi H, Zhou W. Hypoxic microenvironment in cancer: Molecular mechanisms and therapeutic interventions. Signal Transduct Target Ther. 2023;8(1):70. 10.1038/s41392-023-01332-8 PMC9935926 36797231

[20] Cheng Y, Ji Y. Mitochondria-targeting nanomedicine self-assembled from GSH-responsive paclitaxel-ss-berberine conjugate for synergetic cancer treatment with enhanced cytotoxicity. J Control Release. 2020;318:38–49. 10.1016/j.jconrel.2019.12.011 31830542

[21] Kong H, Chu Q, Fang C, Cao G, Han G, Li X. Cu–ferrocene-functionalized CaO_2_ nanoparticles to enable tumor-specific synergistic therapy with GSH depletion and calcium overload. Adv Sci. 2021;8(14):2100241. 10.1002/advs.202100241 PMC8292872 34032026

[22] Garrett DC, Wang LV. Acoustic sensing with light. Nat Photonics. 2021;15(5):324–326.

[23] Wang M, Xue F, An L, Wu D, Sha S, Huang G, Tian Q. Photoacoustic-imaging-guided photothermal regulation of calcium influx for enhanced immunogenic cell death. Adv Funct Mater. 2024;34(19): Article 2311853.

[24] Chen X, Kang R, Kroemer G, Tang D. Organelle-specific regulation of ferroptosis. Cell Death Differ. 2021;28(10):2843–2856. 10.1038/s41418-021-00859-z PMC8481335 34465893

[25] Yu Y, Yan Y, Niu F, Wang Y, Chen X, Su G, Liu Y, Zhao X, Qian L, Liu P, et al. Ferroptosis: A cell death connecting oxidative stress, inflammation and cardiovascular diseases. Cell Death Discov. 2021;7(1):193. 10.1038/s41420-021-00579-w PMC8313570 34312370

[26] Pittet MJ, Michielin O, Migliorini D. Clinical relevance of tumour-associated macrophages. Nat Rev Clin Oncol. 2022;19(6):402–421. 10.1038/s41571-022-00620-6 35354979

[27] Song M, Liu T, Shi C, Zhang X, Chen X. Bioconjugated manganese dioxide nanoparticles enhance chemotherapy response by priming tumor-associated macrophages toward M1-like phenotype and attenuating tumor hypoxia. ACS Nano. 2016;10(1):633–647. 10.1021/acsnano.5b06779 PMC5242343 26650065

[28] Sang Y, Deng Q, Cao F, Liu Z, You Y, Liu H, Ren J, Qu X. Remodeling macrophages by an iron nanotrap for tumor growth suppression. ACS Nano. 2021;15(12):19298–19309. 10.1021/acsnano.1c05392 34783526

[29] Fan P, Zhang N, Candi E, Agostini M, Piacentini M, Francesca B, Pierluigi B, Alessandro M, Giuseppe N, Valentina R, et al. Alleviating hypoxia to improve cancer immunotherapy. Oncogene. 2023;42(49):3591–3604. 10.1038/s41388-023-02869-2 37884747

[30] Wang YN, Wang YY, Wang J, Bai WJ, Miao NJ, Wang J. Vinblastine resets tumor-associated macrophages toward M1 phenotype and promotes antitumor immune response. J Immunother Cancer. 2023;11(8): Article e007253. 10.1136/jitc-2023-007253 PMC10476141 37652576

[31] Rodell CB, Arlauckas SA-O, Cuccarese MF, Garris CA-O, Li R, Ahmed MS, Kohler RA-O, Pittet MJ, Weissleder R. TLR7/8-agonist-loaded nanoparticles promote the polarization of tumour-associated macrophages to enhance cancer immunotherapy. Nat Biomed Eng. 2018;2(8):578–588. 10.1038/s41551-018-0236-8 PMC6192054 31015631

[32] Xu D, Wu L, Yao H, Zhao L. Catalase-like nanozymes: Classification, catalytic mechanisms, and their applications. Small. 2022;18(37): Article e2203400. 10.1002/smll.202203400 35971168

[33] Xu B, Cui Y, Wang W, Li S, Lyu C, Wang S, Bao W, Wang H, Qin M, Liu Z, et al. Immunomodulation-enhanced nanozyme-based tumor catalytic therapy. Adv Mater. 2020;32(33): Article e2003563. 10.1002/adma.202003563 32627937

[34] Koh C-H, Lee S, Kwak M, Kim B-S, Chung Y. CD8 T-cell subsets: Heterogeneity, functions, and therapeutic potential. Exp Mol Med. 2023;55(11):2287–2299. 10.1038/s12276-023-01105-x PMC10689838 37907738

