# Supplementary material for: 2D Titanium-Based Nanozyme Induced a Ferroptosis-like Strategy for Triple-Negative Breast Cancer Therapeutics
Source: Biomater Res. 2026 Sep 21;30:0400. doi: 10.34133/bmr.0400 (PMC13590913; doi:10.34133/bmr.0400)
Supplement: Supplementary file 1 [file bmr.0400.f1.docx]

Figure S1 The XPS spectum of Ti_3_C_2_ reacted with H_2_O_2._

Figure S2 The ROS generation of Ti_3_C_2_ with different concentration

Figure S3 *In vitro* cytotoxic effect of Ti_3_C_2_ nanosheets to 293T cells.

Figure S4 The crystal structure of Ti_3_C_2_ (001) surface.

Figure S5 The immune anti-tumor effects by secreting TNF-α, GzmB, and IFN-γ
